# A novel early screening approach for MCI due to AD based on a “maze” hand-interaction kinetic paradigm

**DOI:** 10.3389/fnins.2026.1788324

**Published:** 2026-06-03

**Authors:** Yuting Tu, Wenbiao Wang, Zhen Yuan, Shuwu Li, Chen Wang, Shouqiang Huang, Zeyu Li, Wenlin Yang, Shengqin Yang, Xun Sun, Yeting Hong, Changxiao Yu, Tong Chen, Kai Li

**Affiliations:** 1 School of Medical Technology and Information Engineering, Zhejiang Chinese Medical University, Hangzhou, China; 2 School of Information Engineering, Hangzhou Medical College, Hangzhou, China; 3 Zhejiang Engineering Research Center for Brain Cognition and Brain Diseases Digital Medical Instruments, Hangzhou Medical College, Hangzhou, China; 4 Centre for Cognitive and Brain Sciences, University of Macau, Taipa, Macau SAR, China; 5 Department of Neurology, The Second Medical Center and National Clinical Research Center for Geriatric Diseases, Chinese PLA General Hospital, Beijing, China

**Keywords:** digital biomarkers, episodic memory, maze paradigm, MCI due to AD, visuospatial and executive functions

## Abstract

**Background:**

Mild cognitive impairment due to Alzheimer’s disease (MCI due to AD) is a crucial stage for the early identification of Alzheimer’s disease (AD), and timely detection at this stage may provide opportunities for earlier intervention and potentially delay disease progression.

**Methods:**

This study proposed a digital early-screening method based on a touchscreen maze hand-interaction kinetic paradigm, which integrates digital biomarkers from the visuospatial/executive and episodic memory domains to support the screening of MCI due to AD. A customized maze task was administered to 40 patients with clinically diagnosed MCI due to AD and 40 healthy controls (HC). Behavioral data were collected, and two categories of digital biomarkers were extracted: (1) visuospatial/executive digital biomarkers, such as task completion time (*VSETT*) and average movement speed (*VSES*); and (2) episodic memory digital biomarkers, such as episodic memory total time (*EMTT*) and number of correct choices (*EMCC*). Significant digital biomarkers were identified through between-group comparisons, and their combined classification performance was evaluated using binary logistic regression and receiver operating characteristic (ROC) analysis.

**Results:**

The integrated digital biomarker model showed promising apparent discriminative performance in the full cohort, with an AUC of 0.899 (95% CI: 0.831–0.967). To reduce potential optimism associated with biomarker selection, model development, and model evaluation within the same dataset, internal validation was performed using full-pipeline repeated stratified five-fold cross-validation with all 16 candidate digital biomarkers entered into the validation procedure and biomarker selection repeated within each training fold. The internally validated model retained good discriminative performance, with a mean cross-validated AUC of 0.842, an empirical 95% interval of 0.779–0.878, an accuracy of 0.783, a sensitivity of 0.772, and a specificity of 0.795.

**Conclusion:**

These findings suggest that the proposed touchscreen maze-based digital assessment method may provide a promising and objective approach to supporting the early screening of MCI due to AD.

## 1 Introduction

Alzheimer’s disease (AD) is the most prevalent neurodegenerative disorder among older adults and is characterized by progressive cognitive decline that gradually compromises daily functioning and independence. It poses a major and growing public health challenge worldwide, with current estimates exceeding 50 million affected individuals—a number projected to rise sharply with global population aging (Cano et al., 2021). With disease-modifying anti-amyloid therapies emerging for early-stage AD, clinical emphasis has shifted toward timely detection, risk stratification, and early intervention. In this context, mild cognitive impairment due to Alzheimer’s disease (MCI due to AD) represents a critical prodromal stage and a key window for therapeutic intervention. Individuals at this stage show a significantly elevated annual conversion rate to dementia (approximately 15% (Xu X. et al., 2024)), underscoring the importance of accurate and accessible screening during this early clinical stage.

The early clinical profile of MCI due to AD is characterized by deficits across multiple cognitive domains rather than impairment in a single isolated function. Among these domains, episodic memory impairment is widely regarded as one of the most characteristic cognitive features of early AD, typically manifesting as difficulty in learning and recalling newly acquired information (Alegret et al., 2022; Gagliardi et al., 2023; Pelgrim et al., 2021) . This deficit is closely associated with early degenerative changes in the medial temporal lobe, particularly the hippocampus and entorhinal cortex, as well as disrupted connectivity with posterior nodes of the default mode network (DMN) (Albertina et al., 2024; Chandra et al., 2025; Ekstrom and Hill, 2023). Compared with semantic memory, which may be influenced by accumulated knowledge and educational background, and working memory, which overlaps substantially with executive control, episodic memory provides a context-specific index of recently encoded event-related information (Seo et al., 2023; Zammit et al., 2025).

In addition to episodic memory decline, impairments in visuospatial and executive functions are also prominent in the early stages of AD. Executive dysfunction typically involves deficits in planning, working memory, cognitive flexibility, and response inhibition, and has been linked to fronto-parietal network abnormalities (Coleman et al., 2023). Visuospatial decline is commonly reflected in spatial orientation, visual integration, and navigation (Li et al., 2023), with associated abnormalities in the posterior parietal cortex, occipitotemporal junction, and dorsal visual pathway (Beyh et al., 2022; Schoonover et al., 2024). These functions are particularly relevant to real-world navigation and visually guided behavior. Because visually guided navigation requires not only spatial perception but also planning, attentional control, and flexible decision-making, visuospatial and executive functions are closely intertwined in navigation-related behavior.

Critically, the co-occurrence of deficits across episodic memory, visuospatial processing, and executive control may provide greater predictive value for progression to dementia than impairment in a single cognitive domain alone (Knopman et al., 2024). Therefore, assessment tools that integrate these cognitive domains may be particularly useful for capturing the heterogeneous but convergent cognitive profile of early AD. By combining a visuospatial-executive module with a landmark-based episodic recognition module, the present paradigm was designed to reflect this multi-domain impairment pattern and to support more sensitive detection of early AD-related cognitive changes. Current standard screening for AD largely relies on brief neuropsychological scales such as the Mini-Mental State Examination (MMSE) and the Montreal Cognitive Assessment (MoCA). However, these tools are limited by subjective scoring, educational and cultural bias, practice effects, and insufficient sensitivity to detect subtle, early cognitive changes (Wang et al., 2024). Although advanced biomarkers from neuroimaging (Leuzy et al., 2025), cerebrospinal fluid analysis (Wang Z. et al., 2025), and blood-based assays (Therriault et al., 2024) show high diagnostic accuracy, their high cost, invasiveness, and limited accessibility constrain their routine use in large-scale community or primary-care screening.

Digital biomarkers, defined as objective and quantifiable behavioral or physiological data collected via digital technologies, offer a promising alternative to bridge this gap (Avram et al., 2020). In AD research, metrics derived from wearable sensors, handwriting dynamics, and eye-tracking have demonstrated potential for early detection by capturing subtle alterations in motor control, kinematics, and visual exploration that are often missed by traditional pencil-and-paper tests (Lin et al., 2023; Oyama et al., 2019; Shah et al., 2025). These approaches are especially valuable because they can quantify task performance continuously and objectively, rather than relying solely on total scores or categorical clinical judgments.

However, existing digital approaches vary in their focus and practicality. Virtual reality (VR) navigation tasks provide high ecological validity for assessing spatial memory and orientation but face barriers related to cost, technical complexity, and potential simulator sickness (Carelli et al., 2011; Machado et al., 2019). Eye-tracking paradigms, while highly sensitive to early visual processing and attentional deficits, are susceptible to performance degradation due to head posture changes during testing—with such errors accounting for approximately 45% of the total variance—and are further affected by calibration and other key factors (Wang X. et al., 2025). Conversely, tablet-based serious games can engage multiple cognitive domains in an engaging format but may lack the kinematic granularity to dissect specific motor planning or execution deficits. Thus, an optimal digital screening approach for early AD should balance ecological validity, cognitive specificity, quantitative precision, and practical feasibility.

Among behavioral paradigms, maze tasks are particularly well-suited for assessing the cognitive profile of early AD, as they naturally engage visuospatial processing, executive planning, and spatial memory within a single ecological task. While traditional mazes, such as the Porteus maze and Morris water maze, face limitations in standardization and practicality (Morris, 1984; Ott et al., 2003; Porteus, 1945), touchscreen-based digital maze tasks offer an advantageous balance. They maintain ecological validity related to real-world navigation (Germine et al., 2019; Iliadou et al., 2021), allow for the automated extraction of rich, high-resolution kinematic data, and are scalable, cost-effective, and easy to administer in clinical settings.

Building on this rationale, the present study developed and internally evaluated a novel digital screening method based on a touchscreen maze hand-interaction kinematic paradigm. We aimed to identify sensitive digital biomarkers across visuospatial/executive and episodic memory domains by comparing task performance between patients with MCI due to AD and cognitively healthy controls (HCs). Furthermore, we descriptively evaluated the combined classification performance of these biomarkers relative to established cognitive screening tools. We hypothesized that this integrated digital approach would provide a more objective, sensitive, and scalable tool for the early detection of MCI due to AD.

## 2 Materials and methods

### 2.1 Participant recruitment and selection

#### 2.1.1 Sample size estimation

The sample size was estimated a priori using G*Power software (version 3.1). For an independent two-tailed t-test comparing two groups (MCI due to AD vs. HC), with an assumed medium effect size (Cohen’s *d* = 0.6), an alpha level (α) of 0.05, and a desired statistical power (1—β) of 0.75, the analysis indicated that a total sample size of 80 participants (40 per group) was required.

#### 2.1.2 Recruitment and study population

A total of 85 participants were initially recruited from the Department of Neurology and the Department of Nuclear Medicine at the Second Medical Center of the Chinese PLA General Hospital. Following screening, 4 individuals were excluded for not meeting the inclusion criteria (two for age outside the 45–80 range, one for a history of major depressive disorder, and one for significant hand tremor precluding valid task completion). Of the remaining 81 participants (41 MCI due to AD, 40 HC) who entered the experimental phase, one participant in the MCI due to AD group withdrew due to a change in health status. Consequently, the final analyzable sample comprised 80 participants (40 per group), matching the a priori sample size estimation.

All participants were native Mandarin speakers. On the day of assessment, each participant completed the Chinese versions of the MMSE, the MoCA, and the maze Hand-Interactive Maze Task in a single session to ensure consistency. Demographic data (age, sex, years of education) and cognitive scale scores were recorded.

The study was conducted in accordance with the principles of the Declaration of Helsinki and was approved by the Medical Ethics Committee of the Chinese PLA General Hospital (Approval No.: S2022-770-02). Written informed consent was obtained from all participants prior to any study procedures. For participants with cognitive impairment, a trained researcher ensured their understanding of the study procedures and consent form using a simplified explanation and confirmation of comprehension.

#### 2.1.3 Diagnostic and grouping criteria

Diagnosis of MCI due to AD was based on (1) the 2011 National Institute on Aging—Alzheimer’s Association (NIA-AA) clinical criteria for mild cognitive impairment, and (2) positive amyloid-β (Aβ) deposition on 11C-PIB PET/CT imaging.

Inclusion criteria for all participants were: (a) age between 45 and 80 years; (b) right-handedness; (c) ability to comprehend task instructions and complete the paradigm; and (d) provision of written informed consent.

Exclusion criteria for all participants included: (a) a history of major psychiatric disorders (e.g., schizophrenia, major depressive disorder); (b) a history of significant traumatic brain injury or other severe systemic/neurological disease; (c) history of alcohol or substance dependence; and (d) any sensory, motor, or other condition that would preclude valid task completion.

### 2.2 Design of “maze” hand-interaction kinetic paradigm

The hand-interactive maze used in this study was generated using a maze-generation platform developed by the University of Michigan, with parameter settings referring to relevant studies on cognitive assessment in neurodegenerative diseases (Nef et al., 2020). To accommodate right-handed operation and avoid visual obstruction (Li et al., 2022), the maze path was uniformly designed to run from the bottom-right to the top-left. The maze structure was designed to follow the criteria for the perfect maze criteria (Bellot et al., 2021), which include: the correct path length exceeds 50% of the total maze path; forward dead ends are aligned with the direction of movement; approximately 40% of the correct path contains visible turns; about 2% of the correct path includes decision points (such as T-junctions and crossroads); and on average, each forward dead end contains about 40% turns and about 1% decision points. The maze design was verified using the hunter maze generation algorithm (Lee et al., 2008) and fully complies with the aforementioned standards.

**FIGURE 1:**
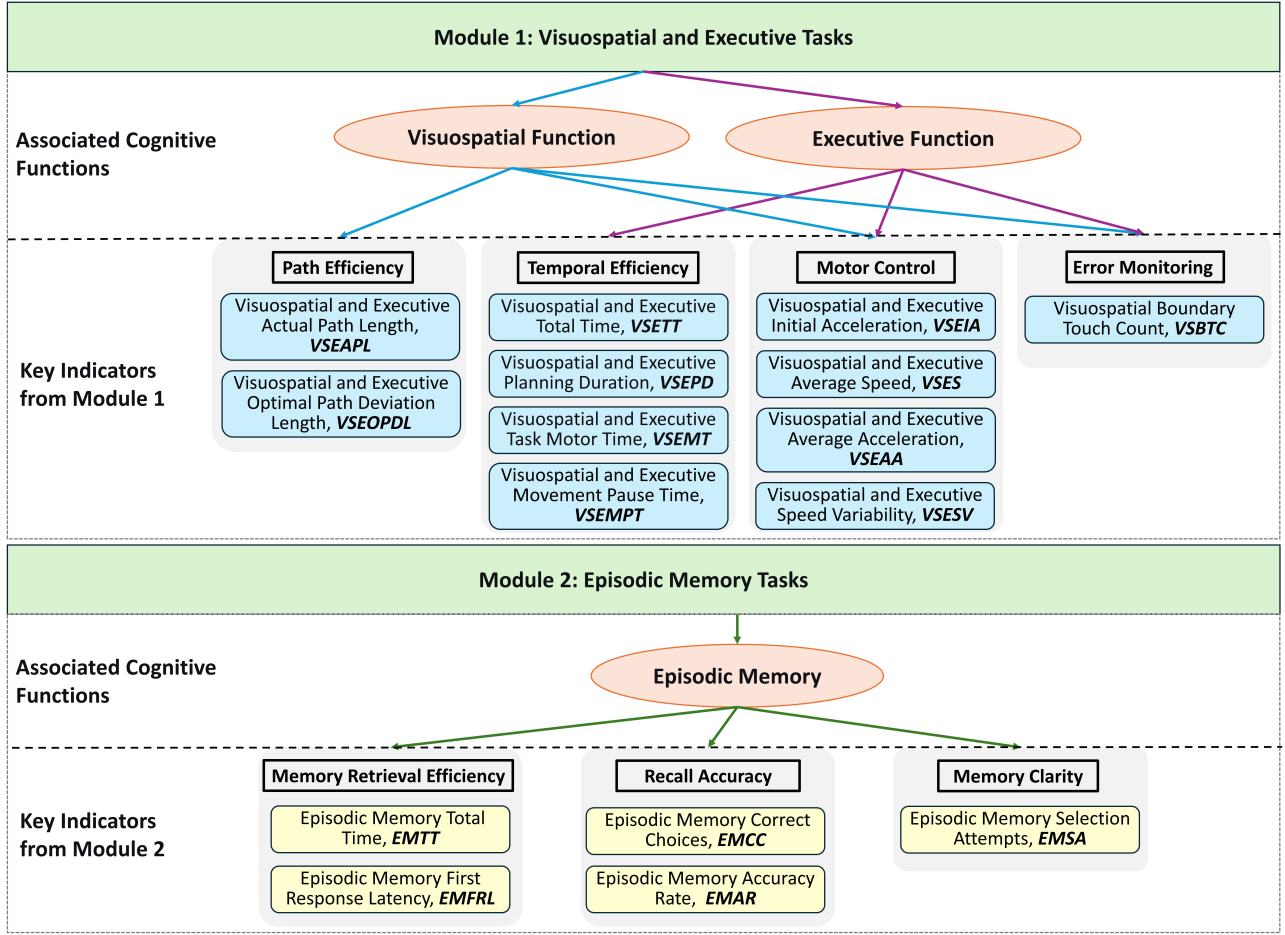
Schematic of the dual-module digital biomarker system and its associated cognitive functions.

The hand-interactive maze task was administered on a touchscreen device and was adapted from a previously validated tool developed by our group to assess executive dysfunction (Huang et al., 2025). To concurrently capture the core cognitive deficits in early MCI due to AD, the original paradigm was redesigned into two integrated, consecutive modules performed within a single ecological task: a visuospatial/executive (VSE) module and a landmark-based episodic recognition module (Barnett et al., 2021; Ekstrom and Hill, 2023), hereafter referred to as the episodic memory (EM) module (see Figure 1 for a schematic).

(1) Visuospatial and Executive (VSE) Module. Participants were instructed to navigate from a predefined start point to the exit of a maze as quickly and accurately as possible. This module assessed spatial processing, planning, and online motor control. Four primary categories of digital biomarkers were extracted from touchscreen interaction logs:
  (a) Path Efficiency, quantified by metrics such as the total path length (*VSEAPL*).
  (b) Time Efficiency, measured by the total task completion time (*VSETT*).
  (c) Motor Control, reflected in the average movement speed (*VSES*).
  (d) Error Monitoring, indicated by the frequency of boundary touches (*VSBTC*).
(2) Episodic Memory (EM) Module. Administered immediately after the VSE module, this part assessed memory for colored landmarks presented in the maze context after navigation completion. Participants were required to memorize these post-navigation landmarks and subsequently identify the target landmarks from an array of distractors:
  (a) Retrieval Efficiency, measured by the latency to the first response (*EMFRL*).
  (b) Recall Accuracy, defined as the number of correct identifications (*EMCC*).
  (c) Retrieval Strategy, inferred from the number of selection attempts (*EMSA*).

This two-module design allows objective and fine-grained quantification of behaviors across cognitive domains that are vulnerable in MCI due to AD, providing a multi-dimensional digital biomarker profile for subsequent analysis.

### 2.3 Data acquisition

The experimental setup consisted of an Intel NUC computer (NUC11PAHi5) and a 17.3-inch interactive display with a resolution of 3,840 × 2,160 pixels (dimensions: 392 × 250 × 10 mm). A touchscreen-based “Maze” hand-interaction kinetics paradigm for digital cognitive assessment was developed in Unity3D. This paradigm was integrated into a human–computer interaction system built with Vue3, which comprised the following modules: a practice module, Assessment Module 1 (visuospatial and executive task), Assessment Module 2 (episodic memory task), and a data acquisition module. Raw interaction data—including participants’ maze trajectory coordinates and task completion times—were recorded as objective performance metrics.

### 2.4 Definition and quantitative analysis of digital biomarkers

The objective data collected from the maze paradigm were processed using the Python platform (version 3.10.14) to extract and quantify digital biomarkers. This included digital biomarkers from Assessment Module 1 and Assessment Module 2. To improve operational consistency, all time- and response-related biomarkers were defined according to task event timestamps and trajectory coordinates recorded by the system.

#### 2.4.1 Visuospatial and executive digital biomarkers

**FIGURE 2:**
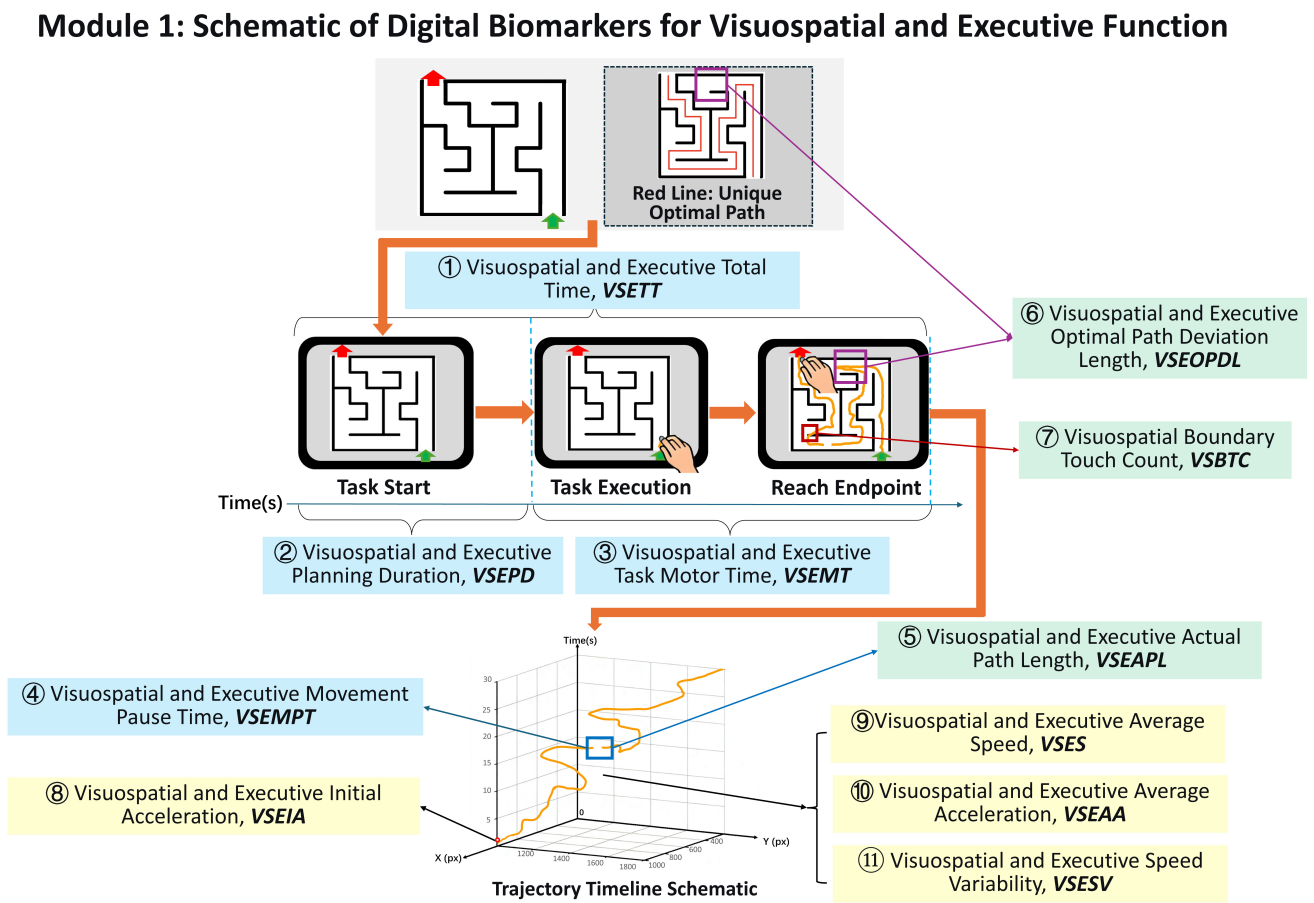
Schematic diagram of visuospatial and executive digital biomarkers.

This section describes the definition and corresponding quantitative calculation methods for each visuospatial and executive digital biomarker (see Figure 2 and Table 1). These metrics serve as core assessment indicators to precisely quantify the visuospatial ability and executive planning capacity demonstrated by participants in Module 1.

**TABLE 1:** Visuospatial and executive digital biomarkers.

Digital biomarker name	Abbreviation	Unit	Definition
Visuospatial and executive actual path length	*VSEAPL*	px	The total length of the movement trajectory drawn by the participant in Module 1.
Visuospatial and executive optimal path deviation length	*VSEOPDL*	px	The cumulative length of the trajectory segments where the participant deviated from the optimal path in Module 1.
Visuospatial and executive total time	*VSETT*	s	The total duration of Module 1 from task onset to successful endpoint touch.
Visuospatial and executive execution time	*VSEET*	s	The interval from the participant’s first valid touch on the starting point to the successful touch on the endpoint in Module 1.
Visuospatial boundary touch count	*VSBTC*	times	The number of times the participant’s finger touched the maze boundaries during Module 1, recorded by the system.
Visuospatial and executive average speed	*VSES*	px/s	The ratio of the total path length to the execution time in Module 1.
Visuospatial and executive speed variability	*VSESV*	/	The variability of movement speed during the participant’s performance in Module 1.
Visuospatial and executive initial acceleration	*VSEIA*	px/s^2^	The acceleration calculated from the first three trajectory sampling points at the beginning of Module 1.
Visuospatial and executive average acceleration	*VSEAA*	px/s^2^	The average acceleration throughout the participant’s performance in Module 1.
Visuospatial and executive planning duration	*VSEPD*	s	The time interval from entering the task to the first touch on the starting point in Module 1, reflecting initial observation and planning.
Visuospatial and executive movement pause time	*VSEMPT*	s	The total duration of all pauses during the participant’s execution of Module 1.

Given a participant’s total visuospatial and executive total time (*VSETT*, *VSETT* < 210 s) and planning duration (*VSEPD*), the execution time (*VSEET*) is calculated using (Equation 1):


V⁢S⁢E⁢E⁢T=V⁢S⁢E⁢T⁢T-V⁢S⁢E⁢P⁢D (1)

In Module 1, the trajectory coordinate sampling frequency (*SF*) was set at 44 Hz (i.e., one sample collected every 0.022 seconds). The total number of trajectory coordinate samples recorded during the assessment was denoted as *I*(*I* = *VSEET* × *SF*). The coordinates of the ith sampled point are represented as (X_i_,Y_i_),(0 < *i* ≤I,*i* ∈ N*). The time interval between any two adjacent sampled trajectory coordinates is *t_i_*, which is calculated using (Equation 2):


$$t_{i}=\frac{1}{S\,F}\left(0<i\leq I-1,i\in N^{{}^{*}}\right)$$ (2)

Visuospatial and executive movement pause time (*VSEMPT*) is calculated by determining whether adjacent trajectory coordinate points are identical, using (Equation 4):


$$j\,u\,d\,g\,e\,\left(i\right)=\left\{ \begin{array}{c} 1\left(X_{i+1}=X_{i}andY_{i+1}=Y_{i}\right)\\ 0\left(X_{i+1}\neq X_{i}orY_{i+1}\neq Y_{i}\right) \end{array}\right.$$ (3)

$$VSEMPT=\frac{\sum_{i=1}^{i=I-1}j\,u\,d\,g\,e\,\left(i\right)}{S\,F}\left(0<i\leq I-1,i\in N^{*}\right)$$ (4)

In Equation 3, *judge*(*i*) is used to determine whether adjacent trajectory coordinate points are identical.

The distance between two adjacent sampled trajectory coordinate points is *D_i_*, calculated as:


$$D_{i}=\sqrt{{\left(X_{i+1}-X_{i}\right)}^{2}+{\left(Y_{i+1}-Y_{i}\right)}^{2}}\left(0<i\leq I-1,i\in N^{*}\right)$$ (5)

In Equation 5, *X*_*i+1*_ is the x-coordinate of the (i+1)th trajectory point, *Y*_*i+1*_ is its y-coordinate, *X_i_* is the x-coordinate of the ith trajectory point, and *Y_i_* is its y-coordinate.

The visuospatial and executive actual path length (*VSEAPL*) is the sum of the distances *D_i_* between all adjacent trajectory coordinate points, calculated using (Equation 6):


$$VSEAPL=\overset{i=I-1}{\underset{i=1}{\sum }}D_{i}\left(0<i\leq I-1,i\in N^{*}\right)$$ (6)

Since the shortest path through the maze (i.e., the optimal decision path for exit) in Module 1 is unique, the region corresponding to this shortest path is defined as φ. Consequently, any task trajectory coordinates sampled outside region φ are designated as erroneous trajectory coordinates (*EX*_i_, *EY*_i_). The distance between two adjacent erroneous trajectory samples is denoted as *ED*_*i*_, which is calculated using (Equation 7):


$$ED_{i}=\sqrt{{\left(E\,X_{i+1}-E\,X_{i}\right)}^{2}+{\left(E\,Y_{i+1}-E\,Y_{i}\right)}^{2}}\left(1\leq i\leq I-1,i\in N^{*}\right)$$ (7)

The Visuospatial and executive optimal path deviation length (*VSEOPDL*) is calculated using (Equation 8):


$$VSEOPDL=\overset{i=I-1}{\underset{i=1}{\sum }}ED_{i}\left(1\leq i\leq I-1,i\in N^{*}\right)$$ (8)

The Visuospatial and executive average speed (*VSES*) is calculated using (Equation 9):


$$V\,S\,E\,S=\frac{V\,S\,E\,A\,P\,L}{V\,S\,E\,T\,T}$$ (9)

We calculate the speed *V_i_* between each pair of adjacent task trajectory coordinate points. Subsequently, the speed *V_i_*, Visuospatial and executive speed variability (*VSESV*), initial acceleration (*VSEIA*), and average acceleration (*VSEAA*) are calculated using (Equations 10, 12–14), respectively:


$$V_{i}=\frac{D_{i}}{t_{i}}\left(0<i\leq I-1,i\in N^{*}\right)$$ (10)

$$\sigma_{V}=\sqrt{\frac{\sum_{i=1}^{I-1}{\left(V_{i}-V\,S\,E\,S\right)}^{2}}{n}}$$ (11)

$$V\,S\,E\,S\,V=\frac{\sigma_{V}}{V\,S\,E\,S}$$ (12)

$$V\,S\,E\,I\,A=\frac{\left|V_{1}-V_{2}\right|}{t_{1}+t_{2}}$$ (13)

$$V\,S\,E\,A\,A=\overset{i=I-1}{\underset{i=1}{\sum }}\frac{\left|V_{i}-V_{i+1}\right|}{t_{i}+t_{i+1}}$$ (14)

In Equation 11, σ_*V*_ represents the standard deviation of the execution speed.

#### 2.4.2 Episodic memory digital biomarkers

**FIGURE 3:**
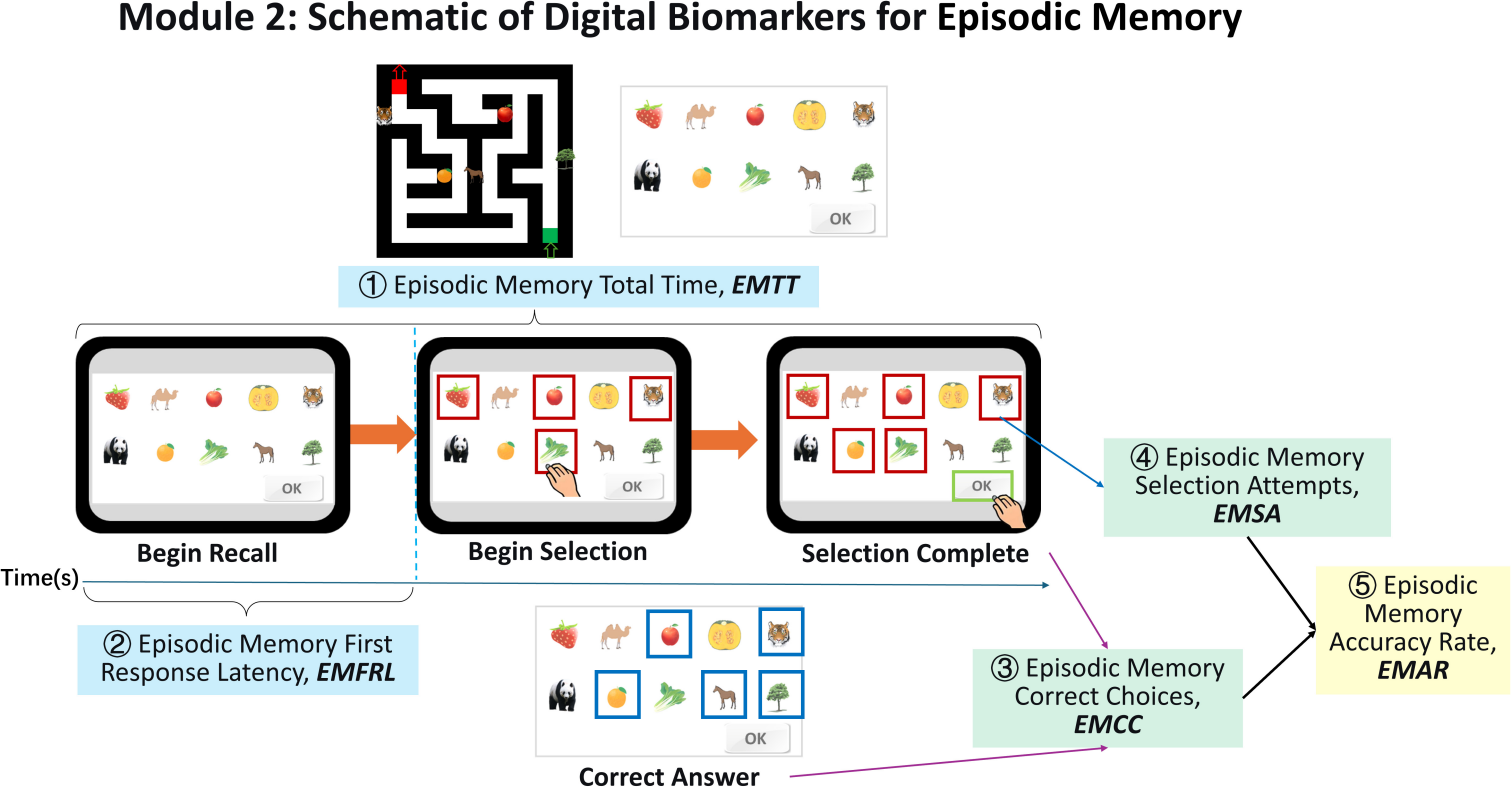
Schematic diagram of episodic memory digital biomarkers.

This section details the definition and corresponding quantitative calculation methods for each episodic memory digital biomarker (see Figure 3 and Table 2). These metrics serve as core assessment indices to reflect the episodic memory ability demonstrated by participants in Module 2.

**TABLE 2:** Episodic memory digital biomarkers.

Digital biomarker name	Abbreviation	Unit	Definition
Episodic memory total time	*EMTT*	s	The total time taken by the participant to complete Module 2.
Episodic memory first response latency	*EMFRL*	s	The time interval from the initiation of Module 2 to the participant’s first selection of a landmark.
Episodic memory correct choices	*EMCC*	count	The number of landmarks correctly identified by the participant in Module 2.
Episodic memory selection attempts	*EMSA*	count	The total number of landmark selection attempts made by the participant in Module 2, regardless of whether the selected landmark was correct or incorrect.
Episodic memory accuracy rate	*EMAR*	(ratio)	The ratio of correct landmark selections in Module 2, calculated as the number of correct choices divided by the total number of selection attempts.

Given the participant’s number of correct episodic memory choices (*EMCC*) and the number of episodic memory selection attempts (*EMSA*), the episodic memory accuracy rate (EMAR) is calculated using (Equation 15):


$$E\,M\,A\,R=\frac{E\,M\,C\,C}{E\,M\,S\,A}$$ (15)

### 2.5 Experimental procedure for the maze hand-interaction kinetic paradigm

**FIGURE 4:**
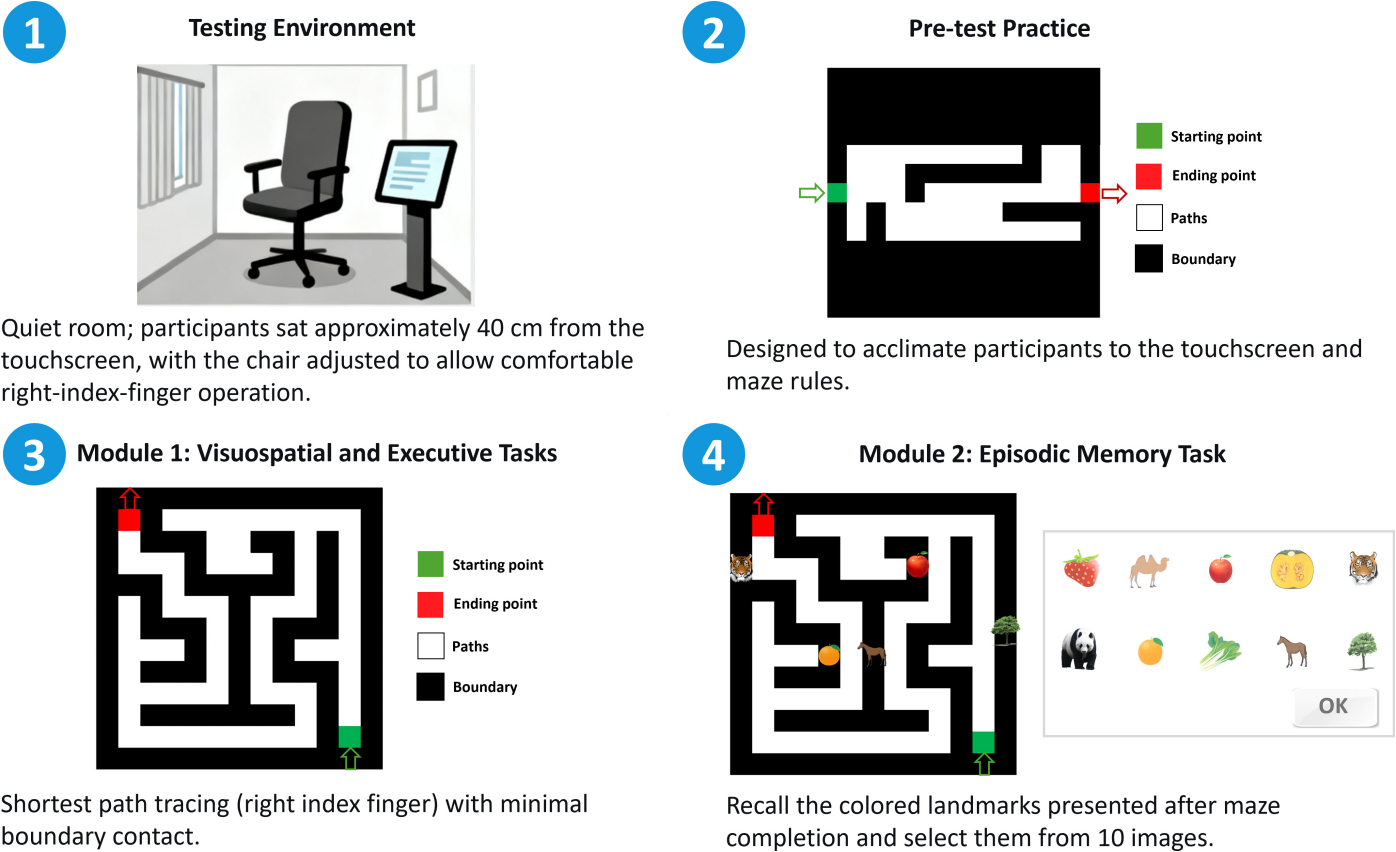
Experimental procedure for the maze hand-interaction kinetic paradigm.

All testing sessions were conducted in a quiet room. Participants sat on an adjustable chair directly facing the interactive display. The chair position was adjusted individually so that the participant’s eyes were approximately 40 cm from the screen and the right index finger could comfortably reach the full touchscreen area. Participants were asked to maintain a stable seated posture during the task. Before the formal experiment began, all participants completed a training trial to familiarize themselves with the task procedure and touchscreen operation. Participants were instructed to use the pad of their right index finger to plan and trace the shortest possible path from a green starting point to a red endpoint on the screen, while avoiding contact with the maze boundaries as much as possible. Ten seconds after reaching the endpoint, a set of colored landmarks was presented on the screen, which participants had 20 se to memorize. Following a 30-s delay after the landmarks disappeared, the recall phase began. Within a set time limit, participants were required to identify and select all previously presented landmarks from an array of options. The specific procedure is detailed in Figure 4.

### 2.6 Data analysis

Primary statistical analyses were performed using SPSS software package version 26.0, and the full-pipeline repeated stratified five-fold cross-validation analysis was conducted using Python. Group differences for categorical data were examined using the Chi-square test. For continuous variables, normality was assessed before group comparisons. Normally distributed continuous data are presented as mean standard deviation and were compared using the independent-samples *t*-test, whereas non-normally distributed continuous variables are presented as median (interquartile range) and were compared using the Mann–Whitney U test.

For the full-cohort between-group comparisons of digital biomarkers, Benjamini–Hochberg false discovery rate (FDR) correction was applied to account for multiple comparisons across the 16 candidate digital biomarkers. Both raw and FDR-adjusted *p*-values were reported, and digital biomarkers with FDR-adjusted *p*0.05 were considered statistically significant in the full-cohort between-group analysis. Digital biomarkers showing significant between-group differences after FDR correction were entered into stepwise binary logistic regression models for exploratory biomarker selection, with the aim of identifying a parsimonious and clinically interpretable set of task-derived digital biomarkers. Predicted probabilities generated from the logistic regression models were used to construct receiver operating characteristic (ROC) curves, and the area under the curve (AUC) was calculated to evaluate diagnostic performance. The evaluation covered three categories: (1) individual visuospatial and executive digital biomarkers; (2) individual episodic memory digital biomarkers; and (3) combined biomarkers from both domains. To examine the potential influence of demographic factors, two additional apparent analyses were conducted in the full cohort: a demographic-only binary logistic regression model including age, sex, and years of education, and an adjusted binary logistic regression model including these demographic variables together with the digital biomarkers selected in the full-cohort exploratory analysis. For other statistical analyses, a two-sided *p*0.05 was considered statistically significant unless otherwise specified.

To address potential optimism due to digital biomarker selection, model development, and model evaluation within the same dataset, internal validation was performed using full-pipeline repeated stratified five-fold cross-validation. Given the modest sample size and the known limitations of data-driven variable selection procedures, this validation strategy was used to further examine the robustness of the selected biomarker model. All 16 candidate digital biomarkers were included in the validation procedure. In each training fold, the distribution of each candidate digital biomarker was assessed. Normally distributed variables were compared between groups using the independent-samples *t*-test, whereas non-normally distributed variables were compared using the Mann–Whitney U test. Digital biomarkers with *p*0.05 in the training fold were selected as predictors. A binary logistic regression model was then fitted using only the training data, and the held-out fold was used exclusively for model evaluation. This five-fold procedure was repeated 100 times with different random splits while preserving the proportion of MCI due to AD and HC participants in each fold. The mean cross-validated AUC and the 2.5th–97.5th percentile interval across repeated cross-validation runs were reported, together with accuracy, sensitivity, and specificity. In addition, a fixed-marker cross-validation analysis based on the digital biomarkers selected in the full cohort was performed as a supplementary analysis.

In the internal validation analyses, the primary cross-validation models were based on digital biomarkers only. In addition, demographic-adjusted sensitivity analyses were performed in both the full-pipeline and fixed-marker cross-validation frameworks, in which age, sex, and years of education were forced into the logistic regression model within each training fold after biomarker selection. These demographic variables were not subjected to univariate biomarker screening but were included as covariates in the model.

## 3 Results

### 3.1 Demographic and clinical characteristics

**FIGURE 5:**
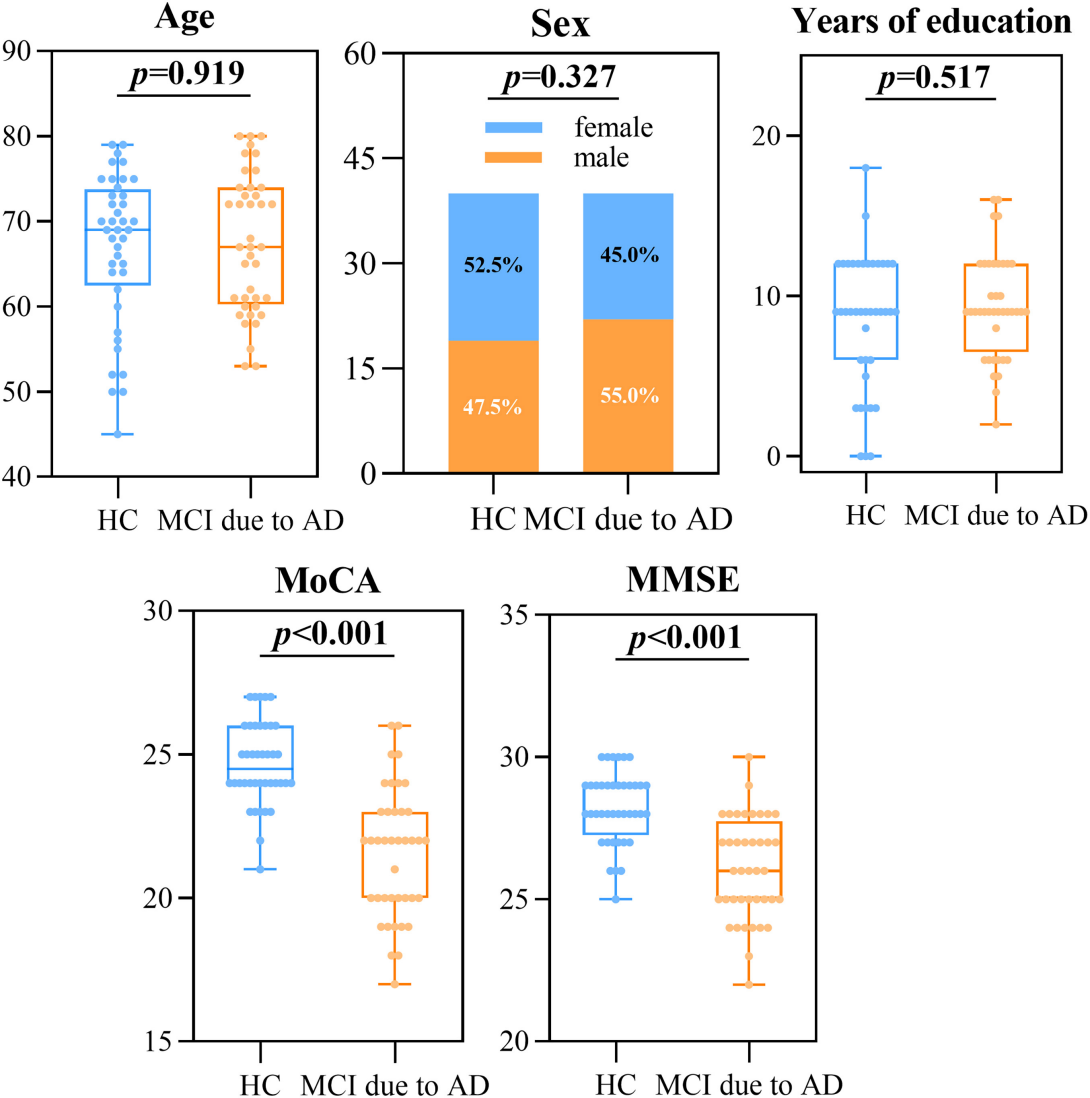
Distribution of demographic and clinical characteristics in the HC and MCI due to AD groups.

The final analyzable sample comprised 80 participants, including 40 patients with MCI due to AD and 40 cognitively normal older adults, who were assigned to the MCI due to AD group and the HC group, respectively. First, we compared the baseline characteristics between the two groups. There were no significant differences between the two groups in age(*p* = 0.919), sex (*p* = 0.327), years of education (*p* = 0.517), MMSE (*p* < 0.001), and MoCA (*p* < 0.001) as shown in Figure 5 and Table 3.

**TABLE 3:** Results of between-group comparisons for demographic and clinical characteristics.

Characteristic	HC Group (*n* = 40)	MCI due to AD Group (*n* = 40)	Statistic(t/Z/χ^2^)	*p*-value
Age, years	69 (11.25)	67 (13.75)	*Z* = −0.101	0.919
Sex (female/male)	21/19	18/22	χ^2^ = 0.450	0.327
Years of education	9.00 (6.00)	9.00 (5.50)	*Z* = −0.649	0.517
MMSE score	28.00 (1.75)	26.00 (2.75)	*Z* = −5.215	**< 0.001**
MoCA score	24.50 (2.00)	22.00 (3.00)	*Z* = −5.800	**< 0.001**

HC, Healthy Control; MCI due to AD, Mild cognitive impairment due to Alzheimer’s disease; MMSE, Mini-Mental State Examination; MoCA, Montreal Cognitive Assessment. Continuous variables are presented as mean ± standard deviation (if normally distributed) or median (interquartile range) (if non-normally distributed). Group comparisons were performed using Independent Samples *t*-test (for normally distributed data), Mann–Whitney U test (reported as *Z* value, for non-normally distributed data), or Chi-square test (for categorical data). The significance level was set at *p* < 0.05. Significant *p*-values are highlighted in bold.

At enrollment, all participants were assessed by clinicians using the MMSE and MoCA scales. Intergroup comparisons revealed that the MCI due to AD group had significantly lower scores than the HC group on both the MMSE (*p* < 0.001) and the MoCA (*p* < 0.001).

### 3.2 Analysis of digital biomarkers

**FIGURE 6:**
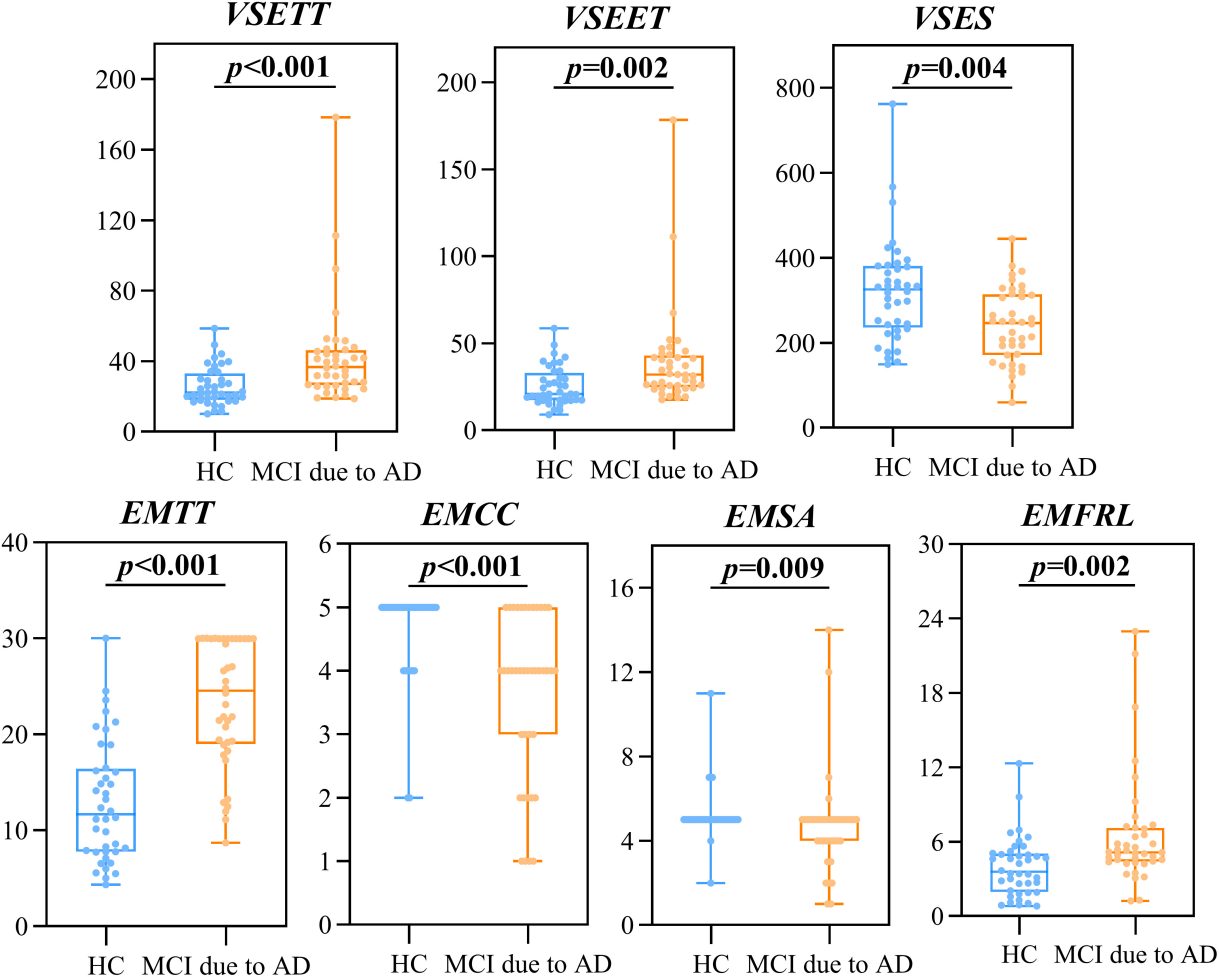
Digital biomarkers showing significant differences between the HC and MCI due to AD groups after Benjamini–Hochberg FDR correction.

We compared visuospatial/executive digital biomarkers and episodic memory digital biomarkers between the HC and MCI due to AD groups. After Benjamini–Hochberg FDR correction across the 16 candidate digital biomarkers, 3 visuospatial/executive biomarkers and 4 episodic memory biomarkers remained significantly different between the HC and MCI due to AD groups. The *VSES* and *EMCC* were significantly lower in the MCI due to AD group than in the HC group, whereas the *VSETT*, *VSEET*, *EMTT*, *EMSA*, and *EMFRL* were significantly higher in the MCI due to AD group. *VSEAPL* showed only borderline significance before correction and did not remain significant after FDR correction. The results of between-group difference tests are shown in Figure 6 and Table 4.

**TABLE 4:** Results of between-group comparisons for digital biomarkers in the HC and MCI due to AD groups.

Digital biomarker	HC group (*n* = 40)	MCI due to AD group (*n* = 40)	*Z*-value	Raw *p-*value	FDR-adjusted *p*-value
Visuospatial and executive digital biomarkers					
*VSEAPL*	6568.11 (958.13)	6758.25 (1684.45)	−1.963	0.050	0.1
*VSETT*	22.36 (14.96)	36.71 (19.92)	−4.099	**< 0.001**	**<0.001**
*VSEPD*	0.02 (0.02)	0.03 (0.02)	−0.818	0.413	0.512
*VSEET*	20.89 (15.64)	31.93 (18.37)	−3.531	**< 0.001**	**0.002**
*VSEOPDL*	0.00 (0.00)	0.00 (1027.38)	−0.957	0.338	0.455
*VSBTC*	2.00 (3.75)	2.00 (5.75)	−0.376	0.707	0.758
*VSES*	326.01 (144.99)	246.61 (142.88)	−3.214	**0.001**	**0.004**
*VSEMPT*	0.50 (1.53)	0.92 (2.04)	−1.617	0.106	0.171
*VSESV*	395.56 (282.86)	405.67 (279.80)	−0.144	0.885	0.889
*VSEIA*	0.00 (2319.12)	918.63 (2490.80)	−1.627	0.104	0.171
*VSEAA*	8705.52 (6129.95)	6516.35 (5504.62)	−1.568	0.117	0.172
Episodic memory digital biomarkers					
*EMTT*	11.68 (8.66)	24.56 (11.01)	−5.543	**< 0.001**	**<0.001**
*EMCC*	5.00 (0.00)	4.00 (2.00)	−4.354	**< 0.001**	**<0.001**
*EMSA*	5.00 (0.00)	5.00 (1.00)	−2.890	**0.004**	**0.009**
*EMAR*	1.00 (0.20)	1.00 (0.20)	−0.749	0.454	0.523
*EMFRL*	3.56 (3.09)	5.15 (2.71)	−3.454	**0.001**	**0.002**

Data are presented as median (interquartile range). HC, healthy control; MCI due to AD, mild cognitive impairment due to Alzheimer’s disease. Group comparisons were performed using the Mann–Whitney U test, with *Z*-values reported. Raw *p*-values indicate the unadjusted significance levels for each digital biomarker. FDR-adjusted *p*-values were calculated using the Benjamini–Hochberg procedure across the 16 candidate digital biomarkers. Digital biomarkers with FDR-adjusted *p* < 0.05 were considered statistically significant and are highlighted in bold.

### 3.3 Apparent ROC analysis for identifying MCI due to AD patients

**FIGURE 7:**
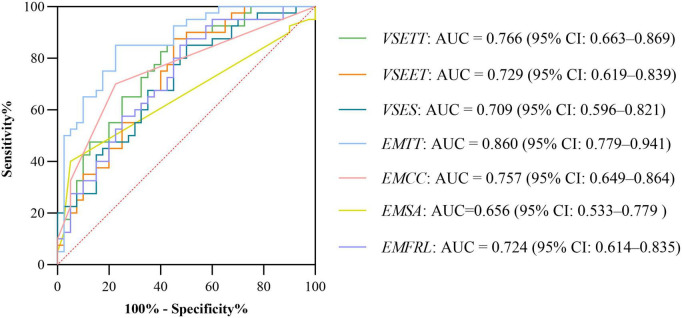
ROC curves, area under the curve (AUC), and 95% confidence intervals (CI) for individual digital biomarkers in screening the MCI due to AD population.

Receiver operating characteristic (ROC) curves were plotted to evaluate the apparent discriminative performance of individual digital biomarkers for identifying patients with MCI due to AD in the entire study sample. Among the seven digital biomarkers that remained significant after FDR correction, the AUC values were 0.766 for *VSETT*, 0.729 for *VSEET*, 0.709 for *VSES*, 0.860 for *EMTT*, 0.757 for *EMCC*, 0.656 for *EMSA*, and 0.724 for *EMFRL* (see Figure 7).

**FIGURE 8:**
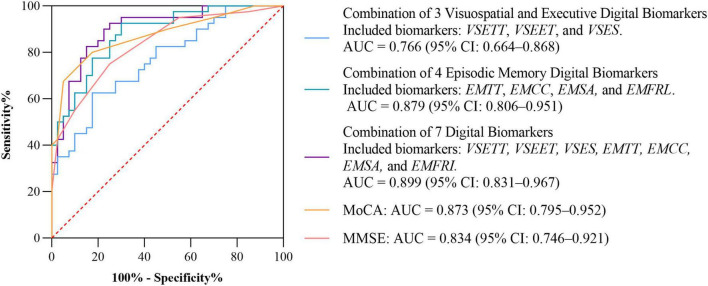
ROC curves, area under the curve (AUC), and 95% confidence intervals (CI) for combined digital biomarkers in screening the MCI due to AD population.

We next evaluated the apparent discriminative performance of models combining these seven digital biomarkers. Specifically, three combined models were examined: a visuospatial/executive model including *VSETT*, *VSEET*, and *VSES*; an episodic memory model including *EMTT*, *EMCC*, *EMSA*, and *EMFRL*; and an integrated model combining biomarkers from both domains. The visuospatial/executive model yielded an AUC of 0.766 (95% CI: 0.664–0.868), whereas the episodic memory model achieved a higher AUC of 0.879 (95% CI: 0.806–0.951). The integrated model showed the highest apparent discriminative performance among the digital biomarker models, with an AUC of 0.899 (95% CI: 0.831–0.967). For descriptive comparison, MoCA and MMSE showed AUC values of 0.873 (95% CI: 0.795–0.952) and 0.834 (95% CI: 0.746–0.921), respectively (see Figure 8).

To further examine whether the apparent discriminative performance of the integrated digital biomarker model was driven by demographic factors, we constructed a demographic-only model including age, sex, and years of education. This model showed limited discriminative performance, with an AUC of 0.542 (95% CI: 0.414–0.670, *p* = 0.516), indicating that demographic variables alone could not effectively distinguish the MCI due to AD and HC groups. By contrast, after further adjustment for age, sex, and years of education, the integrated model including the seven selected digital biomarkers retained high apparent discriminative performance, with an AUC of 0.927 (95% CI: 0.872–0.982, *p* < 0.001). Because this covariate-adjusted apparent AUC was derived from the full cohort, internal validation was further performed to assess whether the model retained similar discriminative performance.

### 3.4 Internal validation of the integrated digital biomarker model

Internal validation using full-pipeline repeated stratified five-fold cross-validation showed that the integrated digital biomarker model retained good discriminative performance. All 16 candidate digital biomarkers were entered into the validation pipeline, and digital biomarker selection was repeated within each training fold. The integrated model achieved a mean cross-validated AUC of 0.842, with an empirical 95% interval of 0.779–0.878. The corresponding accuracy, sensitivity, and specificity were 0.783, 0.772, and 0.795, respectively. A supplementary fixed-marker cross-validation analysis based on the digital biomarkers selected in the full cohort yielded comparable performance, with a mean cross-validated AUC of 0.844 and an empirical 95% interval of 0.790–0.875. The corresponding accuracy, sensitivity, and specificity were 0.783, 0.774, and 0.792, respectively.

In sensitivity analyses with age, sex, and years of education forced into the logistic regression models, the full-pipeline model achieved a mean cross-validated AUC of 0.834, with an empirical 95% interval of 0.761–0.880. The demographic-adjusted fixed-marker model achieved a mean cross-validated AUC of 0.840, with an empirical 95% interval of 0.774–0.878. These internally validated results were similar to those of the corresponding digital biomarker-only models, suggesting that model discrimination was not materially influenced by demographic variables. Accordingly, the digital biomarker-only model was considered the primary model, whereas the demographic-adjusted models were regarded as sensitivity analyses evaluating the potential influence of age, sex, and education. The complete internal validation results are summarized in Table 5.

**TABLE 5:** Apparent and internal validation performance of the integrated digital biomarker model.

Analysis	AUC	95% CI/empirical 95% interval	Accuracy	Sensitivity	Specificity
Apparent full-cohort model	0.899	0.831–0.967	0.838	0.825	0.850
Full-pipeline repeated stratified five-fold CV	0.842	0.779–0.878	0.783	0.772	0.795
Fixed-marker repeated stratified five-fold CV	0.844	0.790–0.875	0.783	0.774	0.792
Full-pipeline CV +age/sex/education	0.834	0.761–0.880	0.776	0.771	0.781
Fixed-marker CV + age/sex/education	0.840	0.774–0.878	0.777	0.773	0.781

AUC, area under the curve; CI, confidence interval; CV, cross-validation. For the apparent full-cohort model, the 95% CI was calculated from the full-sample ROC analysis. For cross-validation analyses, empirical 95% intervals represent the 2.5th–97.5th percentile intervals across repeated cross-validation runs. Full-pipeline cross-validation incorporated digital biomarker selection within each training fold. Fixed-marker cross-validation used the seven digital biomarkers selected in the full cohort. Analyses additionally adjusted for age, sex, and education were conducted as demographic-adjusted sensitivity analyses.

The seven digital biomarkers identified in the apparent analysis based on the entire study sample showed high selection stability across the full-pipeline cross-validation procedure. *VSETT*, *VSEET*, *EMTT*, and *EMCC* were selected in all training folds, while *EMFRL*, *VSES*, and *EMSA* were selected in 99.8, 99.4, and 94.6% of training folds, respectively. These high selection frequencies suggest that the identified biomarkers were consistently selected across repeated training subsets, supporting their relative stability in this cohort.

## 4 Discussion

This study developed and preliminarily evaluated a novel digital assessment tool based on a touchscreen maze hand-interaction kinetic paradigm for the early screening of MCI due to AD. By extracting and integrating multi-domain digital biomarkers, our method showed promising apparent discriminative performance in the entire study sample (AUC = 0.899, 95% CI: 0.831–0.967). A demographic-only model showed limited discriminative performance, whereas the covariate-adjusted integrated model retained high apparent discrimination, suggesting that the observed classification performance was not attributable to demographic variables alone. The main between-group differences in digital biomarkers remained significant after Benjamini–Hochberg FDR correction, suggesting that the selected markers were unlikely to be driven by multiple-testing artifacts. Importantly, after full-pipeline repeated stratified five-fold cross-validation with biomarker selection embedded within each training fold, the integrated model retained good discriminative performance (mean cross-validated AUC = 0.842, empirical 95% interval: 0.779–0.878). A supplementary fixed-marker cross-validation analysis yielded comparable results, and the selected digital biomarkers showed high selection stability across repeated resampling. Demographic-adjusted sensitivity analyses further suggested that this performance was not primarily driven by age, sex, or years of education. Nevertheless, because all validation analyses were based on a modest single-center cohort, the cross-validated AUC should be regarded as a conservative internal estimate of model performance and should be confirmed in larger external cohorts. These findings suggest that the proposed paradigm may provide an objective, efficient, and scalable digital approach for supporting early screening of MCI due to AD.

### 4.1 Deficits in visuospatial and executive functions: behavioral manifestations and neural correlates

The MCI due to AD group exhibited overall inefficiency in Module 1, characterized by a significantly reduced average movement speed (*VSES*) and markedly prolonged total time (*VSETT*) and execution time (*VSEET*). These findings suggest reduced visuomotor-executive efficiency during the maze task, involving online visuospatial processing, path planning, and goal-directed touchscreen movement execution. Such deficits align with documented difficulties in processing spatial cues and inefficient motor planning in early AD (Castegnaro et al., 2023; Smirnov et al., 2022; Ying et al., 2023). Behaviorally, this inefficiency was reflected in the observed kinematic alterations related to task time and movement speed. However, these time- and speed-related biomarkers should not be interpreted as pure measures of executive dysfunction, because successful task performance also depends on visuomotor coordination and general motor execution. In contrast, *VSEAPL* and other error- or acceleration-related features did not remain significant after correction, suggesting that MCI due to AD was more strongly characterized by slowed task execution than by gross path deviation or unstable movement dynamics in this paradigm.

From a neurobiological perspective, successful performance in this maze task may depend on the coordinated function of a network involving the posterior parietal cortex for visuospatial processing, the prefrontal cortex for executive planning and decision-making, and the anterior cingulate cortex for performance monitoring. Early AD pathology may affect these regions (Dugré and Potvin, 2021; Peng et al., 2025; Xu X. et al., 2024; Zuidema et al., 2024), thereby compromising the efficiency of the associated networks. Therefore, the observed decreases in *VSES* and increases in *VSEET* may represent behavioral correlates of reduced visuospatial-executive and visuomotor efficiency, rather than direct evidence of specific network dysfunction. Neuroimaging evidence further supports this link, as the gray matter volume of regions such as the parahippocampal gyrus—a structure critical for spatial processing and path integration—is positively correlated with spatial navigation performance (Shin et al., 2024). Because this study did not include neuroimaging or connectivity measures, these findings should be interpreted as digital behavioral correlates of visuospatial-executive and visuomotor inefficiency; the neural mechanisms remain inferential rather than directly demonstrated.

### 4.2 Altered episodic memory retrieval patterns: behavioral sensitivity and neural substrates

In the Episodic Memory module, MCI due to AD patients demonstrated a pattern consistent with impaired retrieval efficiency. This was characterized by a decreased number of correct choices (*EMCC*), prolonged total task time (*EMTT*) and first response latency (*EMFRL*), and an increased number of selection attempts (*EMSA*). This profile may indicate a slower, more effortful, and less efficient landmark-based episodic recognition process, requiring more time and more selection attempts to retrieve recently encoded visual-spatial information. Among the individual digital biomarkers, *EMTT* showed the highest apparent AUC, suggesting that total retrieval time may be a particularly sensitive behavioral indicator of early AD-related cognitive inefficiency.

The decline in *EMCC* is compatible with the early pathological involvement of the medial temporal lobe (MTL), particularly the hippocampus and entorhinal cortex (Khan et al., 2025). Aβ deposition and tau pathology may lead to synaptic dysfunction and neuronal loss within these structures, thereby compromising memory consolidation and access (Duzel et al., 2022). The prolonged *EMFRL* may reflect a functional decoupling within the prefrontal-hippocampal circuit, which is crucial for initiating and guiding memory retrieval. Reduced neural synchrony (e.g., in theta oscillations) between the medial prefrontal cortex and the hippocampus in MCI due to AD could delay the rapid activation of memory representations (Jayachandran et al., 2023; Srinivasan et al., 2023). Furthermore, declining executive function and processing speed, associated with prefrontal pathology, may contribute to inefficient retrieval strategies and reduced suppression of irrelevant information, thereby slowing the overall retrieval process (Gustavson et al., 2021; Zhou et al., 2024).

Notably, while the final accuracy rate (*EMAR*) did not differ significantly between groups, the significant increase in *EMSA* in the MCI due to AD group may reflect increased retrieval uncertainty, reduced response confidence, or a more effortful recognition process, rather than definitive evidence of a compensatory retrieval strategy. One possible interpretation is that patients made more selection attempts to cope with weakened retrieval confidence or degraded memory traces, thereby maintaining a relatively stable accuracy rate at the cost of greater time and effort. This finding aligns with previous research (Jiang et al., 2025) and underscores a key advantage of digital kinematics: metrics of processing speed (*EMTT*, *EMFRL*) and selection behavior (*EMSA*) may be more sensitive to early, subtle cognitive decline than traditional endpoint accuracy measures alone. Because this study did not include neuroimaging or electrophysiological data, subjective confidence ratings, or explicit strategy assessments, the increased *EMSA* and prolonged retrieval latencies should be interpreted as behavioral markers of retrieval effort or uncertainty; the proposed prefrontal-hippocampal mechanisms remain preliminary rather than direct mechanistic evidence.

### 4.3 Complementary value of multi-domain digital biomarkers for early detection

A central finding of this study is the complementary nature of digital biomarkers derived from the VSE and EM domains. While EM digital biomarkers alone showed good apparent discriminative performance (AUC = 0.879, 95% CI: 0.806–0.951), consistent with the core amnestic phenotype of MCI due to AD and its associated MTL-prefrontal pathology (Shi et al., 2023), VSE digital biomarkers also provided additional discriminative information (AUC = 0.766, 95% CI: 0.664–0.868). This indicates that cognitive impairment in early AD may extend beyond memory to involve functions supported by posterior cortical (Yeh et al., 2025) and fronto-striatal circuits (Park, 2025; Smirnov et al., 2022).

The fusion of both domains yielded the highest apparent classification performance in the entire study sample (AUC = 0.899, 95% CI: 0.831–0.967), suggesting that visuospatial/executive and episodic memory digital biomarkers may provide complementary information for identifying MCI due to AD. Importantly, after full-pipeline repeated stratified five-fold cross-validation, the integrated model retained good discriminative performance (mean cross-validated AUC = 0.842, empirical 95% interval: 0.779–0.878), indicating that the model remained robust after accounting for potential optimism due to digital biomarker selection and model evaluation within the same dataset. This interpretation was further strengthened by the limited discriminative ability of the demographic-only model and by the demographic-adjusted analyses, in which the integrated model retained high apparent performance and showed similar internally validated performance. Although MoCA and MMSE also showed good descriptive discriminative performance, the maze-based digital paradigm provides process-level behavioral features, such as movement speed, response latency, and selection attempts, highlighting its potential as a promising objective tool for early screening of MCI due to AD.

The complementary value of VSE and EM biomarkers may also be interpreted in light of previous network-level findings in early AD. Prior studies suggest that AD-related cognitive impairment is not confined to isolated medial temporal lobe dysfunction but involves distributed alterations across large-scale networks supporting memory, attention, executive control, and visuospatial processing (Albertina et al., 2024; Groen et al., 2022; Jang et al., 2025; Morrone and Minini, 2023; Wen et al., 2023). From this perspective, VSE biomarkers may reflect behavioral manifestations of disrupted visuospatial-executive processing, whereas EM biomarkers may capture impairment in landmark-based episodic recognition (Tozzi et al., 2024; Wan et al., 2024). Their combination may therefore provide a broader behavioral readout of multi-domain cognitive dysfunction in MCI due to AD, which may partly explain the improved discriminative performance of the integrated model. As no neuroimaging, electrophysiological, or connectivity measures were collected, these network-level interpretations are based on prior literature and remain hypothesis-generating.

### 4.4 Limitations and future directions

This study has several limitations. First, its cross-sectional design precludes evaluation of whether the identified digital biomarkers can predict longitudinal conversion from MCI due to AD to AD dementia. Second, although full-pipeline repeated stratified five-fold cross-validation was used, this remains an internal validation strategy based on a modest, single-center sample. Larger independent cohorts are needed to confirm biomarker stability, model generalizability, and performance across demographic subgroups, and to compare this approach with alternative modeling strategies, including penalized regression methods such as LASSO or ridge regression. Third, although the diagnosis of MCI due to AD was supported by clinical criteria and amyloid-PET, other MCI etiologies, such as vascular cognitive impairment or Lewy body disease, were not included. The disease specificity of these biomarkers therefore requires further investigation in clinically heterogeneous MCI populations. Fourth, this study did not include neuroimaging, connectivity measures, fluid biomarkers, or a simple motor baseline task. Accordingly, network-level interpretations should be considered hypothesis-generating, and the potential contribution of general motor slowing to time- and speed-related biomarkers cannot be fully excluded. Future studies should extend validation to larger, multi-center cohorts, examine longitudinal prognostic value, integrate multimodal biomarkers to clarify brain–behavior relationships, and include simple motor control conditions to improve interpretability.

## 5 Conclusion

In conclusion, this study presents an internally validated digital screening approach based on a touchscreen maze paradigm that quantifies subtle visuospatial/executive and landmark-based episodic recognition deficits in MCI due to AD. By integrating multi-domain digital biomarkers, the tool demonstrated promising discriminative performance and may provide objective and complementary information to conventional paper-and-pencil cognitive screening tests. Its advantages, including objectivity, rapid administration, and low cost, suggest potential utility as an early screening tool for MCI due to AD in community and clinical settings. Future studies with larger, multi-center, and independent cohorts are needed to externally validate the proposed model and further establish its clinical applicability.

## Data Availability

The raw data supporting the conclusions of this article will be made available by the authors, without undue reservation.
